# Estrogen affects the negative feedback loop of PTENP1-miR200c to inhibit PTEN expression in the development of endometrioid endometrial carcinoma

**DOI:** 10.1038/s41419-018-1207-4

**Published:** 2018-12-18

**Authors:** Ruichao Chen, Minfen Zhang, Wenya Liu, Hui Chen, Tonghui Cai, Hanzhen Xiong, Xiujie Sheng, Shaoyan Liu, Juan Peng, Fang Wang, Hao Chen, Wanrun Lin, Xuehu Xu, Wenxin Zheng, Qingping Jiang

**Affiliations:** 1 Department of Pathology, The Third Affiliated Hospital, Guangzhou Medical University, 510150 Guangzhou, China; 2 Key Laboratory of Major Obstetric Diseases of Guangdong Province, The Third Affiliated Hospital, Guangzhou Medical University, 510150 Guangzhou, China; 3 Department of Pathology, University of Texas Southwestern Medical Center, Dallas, TX75390 USA

## Abstract

Endometrial carcinoma is one of the most common malignancies in the female reproductive system. It is well-known that estrogen plays an important role in the pathogenesis of endometrioid endometrial carcinoma (EEC), and induces the cancer suppressor gene PTEN deletion. However, how estrogen affects PTEN expression remains unknown. In the present study, we found in 40 EEC specimens, miR-200c level was higher in most cancer areas than that in the adjacent normal endometrium, while PTEN and PTENP1 were lower. Moreover, the expression of PTEN/PTENP1 and miR-200c also showed a converse relationship in EEC cell lines. In addition, we demonstrated that miR-200c bound directly to PTEN and PTENP1, and PTENP1 could reverse miR-200c inhibition function to PTEN using a dual-luciferase reporter and RNA binding protein immunoprecipitation (RIP) assays. Next, 17β-estradiol (E2) treatment could improve miR-200c and drop the PTEN level, which caused a consequential increase of the phospho-PI3K-AKT pathway genes. When we stably knocked down estrogen receptor α (ERα) expression in the EEC cell line, the effects of E2 on miR-200c and PTEN declined. In addition, it was demonstrated that E2 might modulate cell proliferation, migration and invasion relying on the expression of miR-200c. Taken together, it can be concluded that estrogen improves the miR-200c level by combining with ER, PTENP1 and PTEN could be inhibited by miR-200c, and then activate the PI3K-AKT pathway. This work provided a new mechanism of EEC development and a new potential therapeutic target.

## Introduction

According to the American Cancer Society^1^, endometrial carcinoma (EC) is the most common gynecological malignancy in US. In China, the morbidity of EC gradually increased and became the second most productive gynecological malignancy just behind cervical carcinoma^2^. EC is divided into two types: type I, hormone-dependent EEC and type II, hormone-independent cancer, such as serous adenocarcinoma^3,4^.

Estrogen is a major risk factor for EEC, providing the primary proliferative signal in the endometrium through ER (estrogen receptor)^5^. Extensive experimental studies focused on the mechanisms of estrogen-driven endometrial carcinogenesis and found that estrogen urges a decline in a famous cancer suppressor gene: phosphatase and tensin homolog deleted on chromosome ten (PTEN)^6,7^.

EEC has been known to be associated with mutations or deregulation of PTEN, PI3K/AKT, K-ras, and mismatch repair gene^8^. PTEN is located on chromosome 10q23.3 in the human genome. In normal physiological conditions, PTEN levels in the endometrium fluctuate with the menstrual cycle^9^, suggesting that PTEN is regulated by hormones. PTEN competes with the phosphoinositide 3-kinase (PI3K)/Akt activity and growth pathway^10,11^. Decreased PTEN activity consequently increases AKT signaling, resulting in increased cell proliferation and inhibited apoptosis and resistance of hormonal therapy^12,13^. In EEC pathogenesis, estrogen was found to inhibit PTEN expression through extensive experimental studies^14–16^. However, it is maintained a poorly understood mechanism, which the present study focused on.

In recent years, pseudogene PTENP1, has been reported to regulate the levels of PTEN by competing for miRNAs. The sequence of PTENP1 differs from the PTEN sequence by only 18 mismatches based on the coding region, one of which leads to the elimination of the initiation codon^17^. The upstream region of PTENP1 is highly homologous to PTEN (almost 95% identical), which led to the hypothesis that PTEN-targeted microRNAs could be PTENP1-targeted as well^18^. This hypothesis was confirmed in prostate, colon, and renal cell carcinoma in which PTENP1 binds to and competes with miRNAs to inhibit PTEN expression^17–19^. However, though PTENP1 inactivity is the important mechanism in EEC, there are no related reports^20^.

Many microRNAs have been shown involved in the development of malignancies, including miR-200c. MiR-200c plays an important role in various cancers such as gastric, bladder, and ovarian cancer^21–23^. In the previous studies, we found that miR-200c expression was significantly upregulated in EEC^24,25^. This result is also consistent with other literature^26,27^. It can be considered that miR-200c plays a carcinogenic role in EEC. It was speculated that miR-200c and PTENP1 could affect PTEN expression by a competing mechanism.

In this study, we demonstrated that miR-200c bound directly to PTEN and PTENP1, respectively. E2 treatment verified that both miR-200c and PTEN were regulated by estradiol and showed inverse expression. E2 stimulation increased miR-200c expression and decreased the PTEN level, which caused consequent increases in the phospho-AKT-PI3K pathway, promoted cell proliferation and inhibited apoptosis. Stably knockdown and overexpress ERα in different EEC cell lines shows that the E2 plays its role in the negative feedback regulations of PTENP1-mi200c-PTEN through combination with ERα.

## Materials and methods

### Patients and clinical samples

Forty fresh EEC samples, including cancer and matched adjacent normal tissues, were obtained from Third Affiliated Hospital of Guangzhou Medical University between 2014–2017. The tissues were flash-frozen in liquid nitrogen for 5 min after surgical resection and then stored at −80 °C until RNA extraction.

All the patients provided informed consent, and the study was approved by the ethics committee of Third Affiliated Hospital of Guangzhou Medical University.

### Affymetrix microarray and probe re-annotation

The microRNA expression profiles of endometrial cancer (GSE35794) were downloaded from Gene Expression Omnibus (http://www.ncbi.nlm.nih.gov/geo/), which is based on the Affymetrix Human Genome U133 Plus 2.0 Array platform. Then, the TEX texts (GPL10850) were downloaded from the GEO public data platform, to find the probe number of the hsa-miR-200c gene in GSE35794.

### Cell culture and E2 treatment

Four human EEC cell lines (RL-952, Ishikawa, HEC-1B, and JEC) were ordered from ATCC and maintained in RPMI 1640 (Corning, USA) or Dulbecco’s Modified Eagle’s Medium (DMEM; Corning, USA) supplemented with 10% fetal bovine serum (FBS; Gibco, USA) and 50 µg/ml of penicillin and streptomycin. All cells were grown in 5% CO_2_ at 37 °C.

17β-estradiol (E2, Sigma, USA) was diluted in 100% ethanol and used at a final concentration of 10 nM. The cell were cultured in phenol-red free medium with 5% charcoal-stripped FBS with estrogen, same amount of ethanol was added to the control wells.

### Plasmid construction and cell transfections

The reporter promoter or interference plasmids of PTEN and PTENP1 were synthesized by Yingxin (Guangzhou, China) and named pcDNA3.1-PTEN, pcDNA3.1-PTENP1, RNAi-PTEN, RNAi-PTENP1. Meanwhile, their empty vector, pcDNA3.1(+) and pCDH-CMV-MCS-EF1-copGFP-T2A-Puro were separately used as the control plasmid. Ten micrograms of the PTEN or PTENP1 vector was transfected into EEC cells using Lipofectamine 3000 (Invitrogen, USA) according to the manufacturer's instructions.

The miR-200c mimic and inhibitor primer (Supplementary Table 1), which were synthesized by Ribobio (Guangzhou, China), was transfected to ECC cells using riboFECT™ (Ribobio, Guangzhou, China) according to the manufacturer’s instructions.

### Generation of stable cell line

According to the target gene, we obtained the estrogen receptor alpha(ESR1) sequence to synthesize the shRNA-ER and the Promoter-ER vector (Yingxin, Guangzhou, China). Lentivirus was transfected of the above constructs with packaging plasmids into RL-952 and JEC cell lines, respectively. After 72 h of infection, the cells that not effectively infected were killed by 10 µg/ml of puromycin. The stable cell lines were finally obtained under the maintenance of the puromycin drug and verified by the generation of green fluorescent protein (GFP) using the fluorescence microscope (Supplementary Figure 1).

### RNA extraction and quantitative real-time PCR (qRT-PCR)

Total RNA was isolated using TRIzol reagent (Takara, Japan). Isolated RNA (1 µg) was reverse transcribed into cDNA with a Reverse Transcription Kit (Takara, Japan) and subjected to qRT-PCR using Power SYBR green (Takara, Japan) according to the manufacturer’s instructions. The miR-200c specific primers and U6 were synthesized by Ribobio (Guangzhou, China). QRT-PCR cycling conditions were 95 °C for 30 s, 40 cycles at 95 °C for 5 s, 60 °C for 30 s. Beta-actin acts as an internal reference except miR-200c. The qRT-PCR reactions were performed using an ABI Step One Plus instrument (Applied Biosystems, Foster City, CA). All the primer sequences used are listed in Supplementary Table 1.

### Dual-luciferase reporter assay

The sequence of PTEN or PTENP1 in the pcDNA3.1 plasmids were designed and amplified by PCR and sub-cloned into the psiCheck2.0 vector to luciferase reporter assay. The resulting constructs were named psi-PTEN-WT, psi-PTEN-Mut, psi-PTENP1-WT, and psi-PTENP1-Mut. Briefly, RL-592 cells were incubated in 24-well plates and co-transfected with 100 ng of psicheck 2.0 luciferase vectors containing the WT or Mut, PTEN or PTENP1, and miR-200c mimics or NC according to the experimental groups. The Dual-Luciferase Reporter Assay (Promega, CA) was performed according to the manufacturer's instructions. All the transfection experiments were performed in triplicate.

### Western blot analysis

Cells were lysed using RIPA buffer (Beyotime, P.R. China) containing PMSF. Equal aliquots of proteins were separated by 8% SDS-PAGE and then electro-transferred to PVDF membranes (Millipore, USA). Subsequently, the membranes were blocked and probed with the indicated primary antibody overnight at 4 °C. Afterward, membranes were incubated for 2 h with a secondary antibody. Signal was observed using chemiluminescence assays (Pierce, USA), and proteins were detected and quantified using ChemiDoc-XRS+ (Bio-Rad, CA). Antibodies against PTEN, AKT, phospho-AKT, PI3K, phospho-PI3K, ZEB1, and ESR1 were purchased from Cell Signaling Technology (Shanghai, China).

### Cell proliferation assay

The RL-952 cell was used for the CCK-8 assay. After transfection for 24 h, we planted approximately 7 × 10^3^ cells in 96-well plates and cultured for 1, 2, 3, or 4 days, respectively, before added 10 ml of CCK-8 reagent (DOJINDO, Japan). Then, the cells were re-placed in an incubator for 2 h. Finally, the cells were placed in a Thermomax microplate reader to measure the OD value at 450 nm. All experiments were repeated three times, and the final OD was the mean of the three measurements.

### Apoptosis and cell cycle assays

Apoptosis and the cell cycle were detected and analyzed by flow cytometry in a BD AccuriC6 (BD Biosciences, San Jose, CA) equipped with FlowJo 7.6 software (BD Biosciences, San Jose, CA). The Annexin VePE/7AAD Apoptosis Kit (Multsciences, China) was performed following the manufacturer's instructions, and CellQuest software (BD Biosciences, USA) was used to quantify apoptosis. For the cell cycle assays, the cells were stained with PI using a CycleTEST™ PLUS DNA reagent kit (BD Biosciences, USA). All experiments were performed in triplicate.

### Cell migration and invasion assay

Migration and invasion assays were performed in triplicate using migration chambers (8-mm pore size, Costar) with Matrigel (BD Biosciences). RL-952 cell line was transfected for 24 h in serum-free medium before seeded into the upper chambers of transwells, while the lower chambers filled with medium containing 10% charcoal-stripped FBS. The cells were placed in incubator for several hours, fixed and stained with 0.1% crystal violet. Cells on the lower surface were photographed, and five random fields of cells were counted.

### RNA binding protein immunoprecipitation assay (RIP)

Antibody against Ago2 was purchased from Abcam, # ab32381. AGO2-RNA mixture was obtained using a RIP kit (Millipore, USA) following the manufacturer's instructions. The eluted RNA was reverse transcribed into cDNA with a Reverse Transcription Kit (Takara, Japan) and analyzed by qPCR using SYBR green (Takara, Japan).

### Immunohistochemistry staining (IHC)

Immunohistochemistry was used to determine the protein expression patterns of PTEN (Abcam, USA) in the 40 EEC samples. The experimental sections were visualized after stained using DAB, counterstained with hematoxylin, and mounted in neutral gum. The tissues were then analyzed using a bright-field microscope.

### Statistical analyses

Data were expressed as the mean ± standard deviation of at least three separate experiments. All the tests were two-tailed, and an analysis of variance (ANOVA) for functional analyses was performed. A *P* value < 0.05 was considered statistically significant. Integration of the data was carried out using GraphPad Prism software for windows, version 5.00 (San Diego, CA, USA).

## Results

### Inverse expression of PTEN or PTENP1 and miR-200c in EEC tissue samples and cell lines

**Fig. 1:**
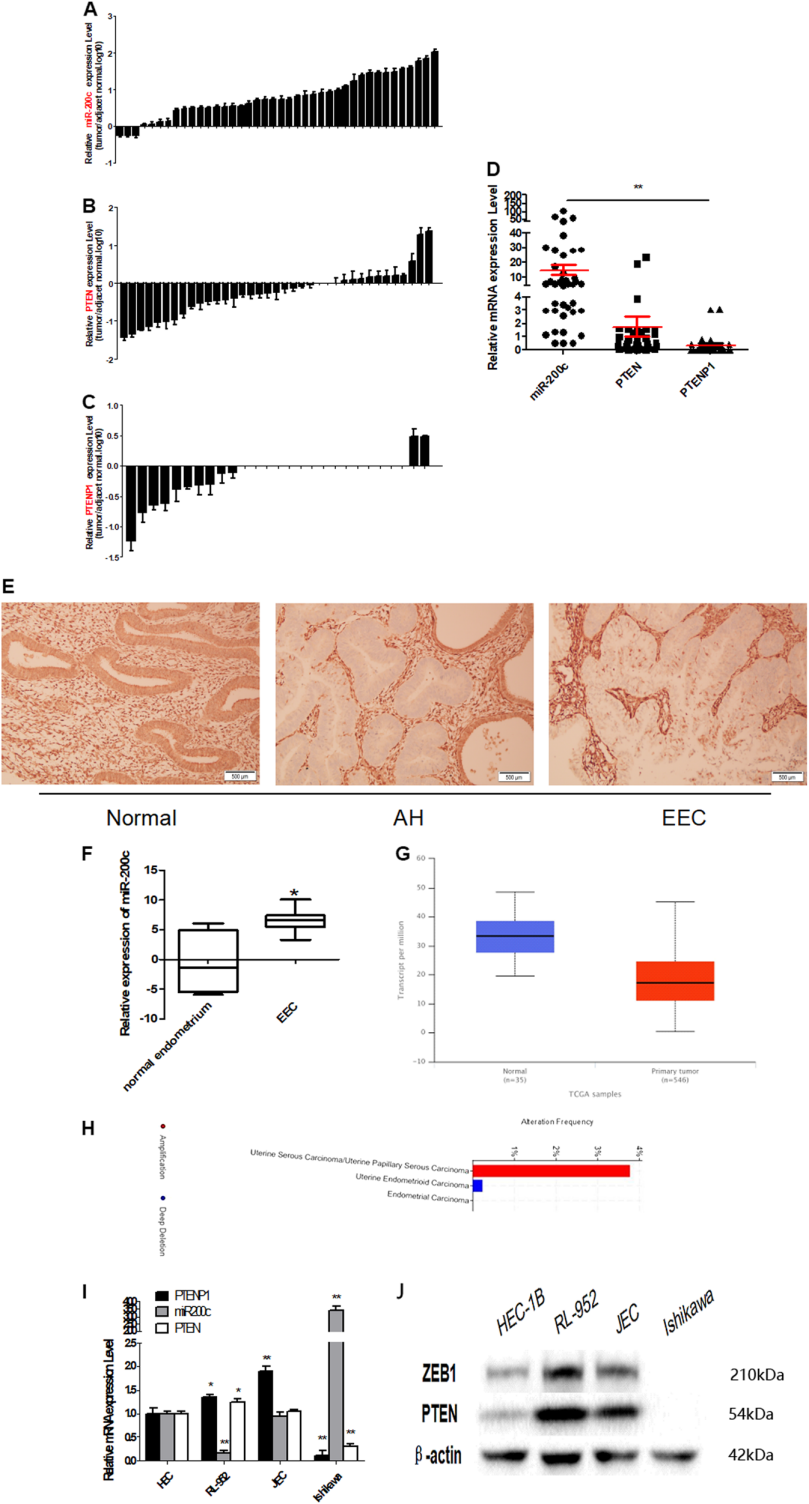
Inverse expression of PTEN or PTENP1 and miR-200c in EEC tissue samples and cell lines. **a** In 37 of 40 EEC samples (92.5%), miR-200c was expressed at higher levels than that in adjacent normal tissues. **b** In 25 of 37 EEC samples (67.6%), PTEN was expressed at lower levels than in adjacent normal tissues. **c** In 25 of 27 EEC samples (92.6%), PTEN was expressed at lower levels than in adjacent normal tissues. **d** miR-200c, PTEN, and PTENP1 expression in 40 cases were analyzed, and showed there was a statistically correlation between their expression. **e** Immunohistochemistry showed that PTEN was lost in most complex atypical hyperplasia and cancer tissues. **f** GEO database was used to analyze the expression of miR-200c in EEC. **g** The expression of PTEN in 546 cases of endometrial carcinoma were analyzed in TGCA database by http://ualcan.path.uab.edu/analysis.html. **h** TGCA database was used to analyze the PTENP1 expression. **i**–**j** In four EEC cell lines the expression of PTEN, PTENP1, and miR-200c was detected by qRT-PCR and western blot. There was an opposite relationship between miR-200c and PTENP1/ PTEN. **P < 0.01

First, we measured PTEN, PTENP1, and miR-200c expression in 40 cases of EEC and their adjacent normal tissues using qRT-PCR. The results found that, compared with the normal tissues, miR-200c expression was significantly higher in EEC tissues (92.50%, 37/40), and the expression of PTEN and PTENP1 was lower (PTEN, 67.6%, 25/37, and PTENP1, 92.6%, 25/27), though there were three cases in which expression of PTEN was negative in the both cancer and adjacent normal tissues, and in 13 cases PTENP1 were neither expressive in the both areas (Fig. 1a–d). Then, PTEN immunohistochemistry was applied to endometrial specimens including normal endometrium, atypical hyperplasia (AH), and EEC, the results were consistent with qRT-PCR (Fig. 1e, Supplementary Table 2). Afterward, we analyzed the expression of miR-200c and PTEN in EEC using the GEO database and TGCA database, respectively. We found that the expression level of miR-200c and PTEN in EEC was consistent with our results. The level of miR-200c was also higher and PTEN was lower in EECs than that in normal tissues (Fig. 1f, g). However, the low expression of PTEN seems unrelated with clinical grades and stages, age and race, etc. (Supplementary Figure 2A–D). Additionally, PTENP1 was presented deletion in EEC, compared to the amplification in serous endometrial carcinoma (Fig. 1h).

We detected the expression of PTEN, PTENP1, and miR-200c in four EEC cell lines (HEC-1B, RL-952, JEC, and Ishikawa) using qRT-PCR and western blot. Some reports demonstrated that the miR-200 family could regulate the EMT process through targeting and downregulating the expression of ZEB1 and ZEB2, including miR-200c^28–30^. We also demonstrated that the expression of miR-200c and ZEB1 protein was opposite in EEC^24^. Therefore, ZEB1 protein was used in the following experiments to reflect the miR-200c expression in the western blot. The results verified that the miR-200c expression was the highest in Ishikawa, while PTENP1 and PTEN was the lowest in this cell line. On the countrary, in RL-952, the expression of PTEN and PTENP1 was the highest, while miR-200c the lowest. In JEC, the difference of PTENP1 and miR-200c was significant, though the expression of miR-200c and PTEN was not so obviously different. In HEC-1B, their exprssion were similar (Fig. 1i, j).

Combined with the experimental results of the EEC clinical samples and cell lines, it was suggested that the expression of PTENP1, PTEN, and miR-200c in EEC was inversely related.

**Fig. 2:**
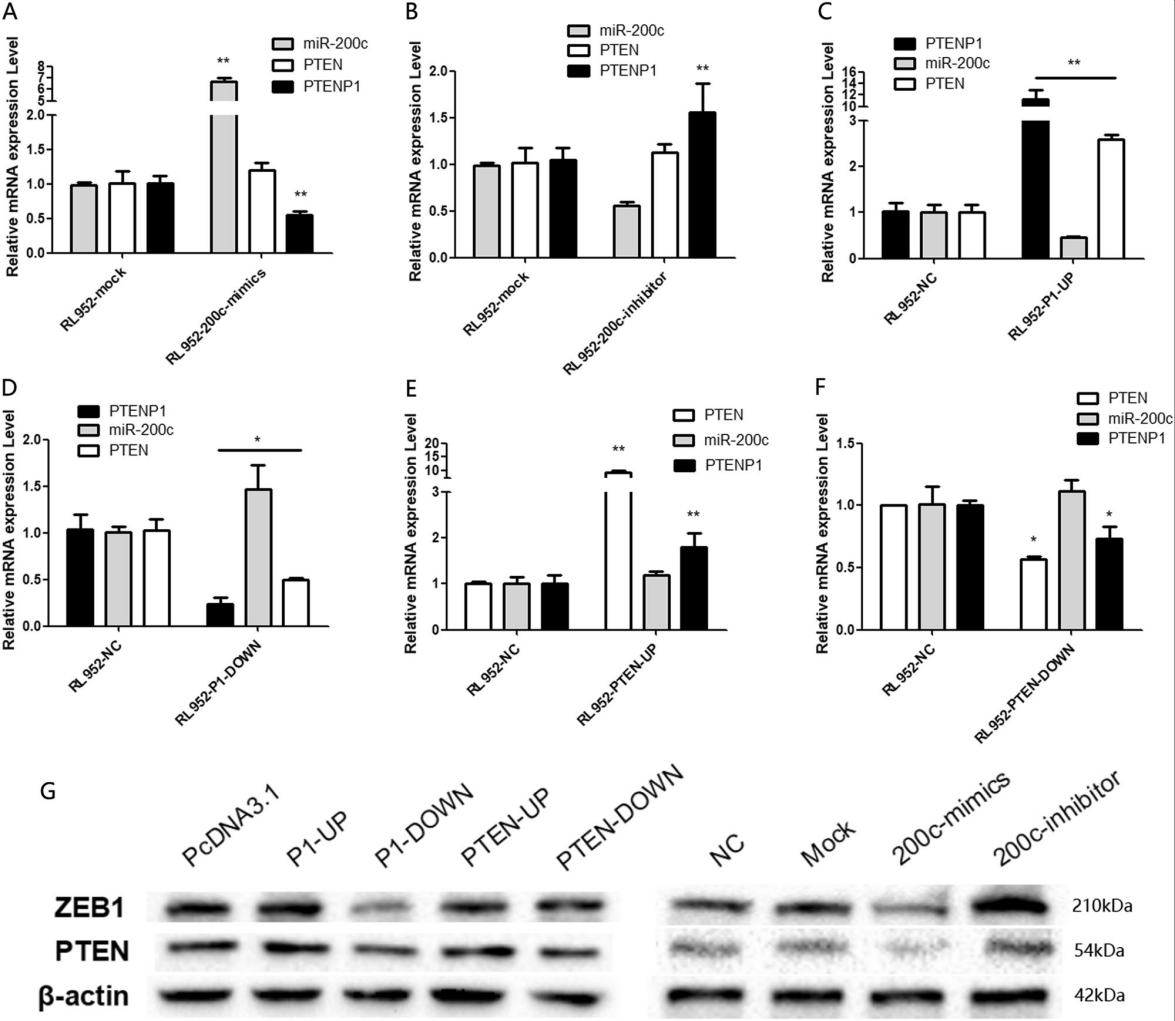
The relationship between miR-200c, PTENP1, and PTEN in EEC cell lines. **a** PTENP1 was downregulated by transfecting RL-952 cells with miR-200c mimics, when PTEN RNA was not significant changed. **b** PTENP1 was upregulated in RL-952 cells when miR-200c expression was inhibited, when PTEN RNA was not significantly changed. **c** PTEN was upregulated and miR-200c was downregulated when PTENP1 expression was increased in RL-952 cells. **d** PTEN was downregulated and miR-200c was upregulated when PTENP1 expression was inhibited in RL-952 cells. **e**, **f** The expression of PTENP1 increased or decreased after PTEN overexpression or interference in RL-952 cells, but there was no significant change in miR-200c expression. **g** the expression of PTEN protein increased or decreased by transfecting RL-952 cells with miR-200c-inhibitor of miR-200c-mimics

To further examine the relationship between miR-200c, PTENP1 and PTEN, RL-952 cells were transfected with miR-200c mimic. The expression of PTENP1 and PTEN protein was decreased after miR-200c mimic transition, but there was no significant change in the expression level of the PTEN RNA. Conversely, transfection of the miR-200c inhibitor into RL-952 cells resulted in increased PTENP1 RNA and PTEN protein expression (Fig. 2a, b, g). Meanwhile, when pcDNA3.1-PTENP1 was transfected into RL-952 cells, miR-200c expression was decreased and PTEN protein increased (Fig. 2c). In contrast, while PTENP1 was downregulated, the expression of miR-200c was overexpressed and PTEN protein was decreased (Fig. 2d). However, when RL-952 cells were transiently transfected with overexpression of PTEN or interfered plasmid, there was no significant change in miR-200c expression, but the expression of PTENP1 increased or decreased after PTEN overexpression or interference in the cells (Fig. 2e, f). HEC-1B and JEC were applied the same experiments, the similar results were gotten, which was shown in supplementary Figure 3 for HEC-1B, and not presented for JEC.

These results suggest that miR-200c inversely regulates PTENP1 and PTEN expression, and PTENP1 inversely regulates miR-200c expression when positively regulated by PTEN. Moreover, between PTENP1 and miR-200c, there was a negative feedback, and between PTENP1 and PTEN, a positive feedback. However, PTEN could not regulate miR-200c.

### PTENP1 and PTEN are target genes of miR-200c

**Fig. 3:**
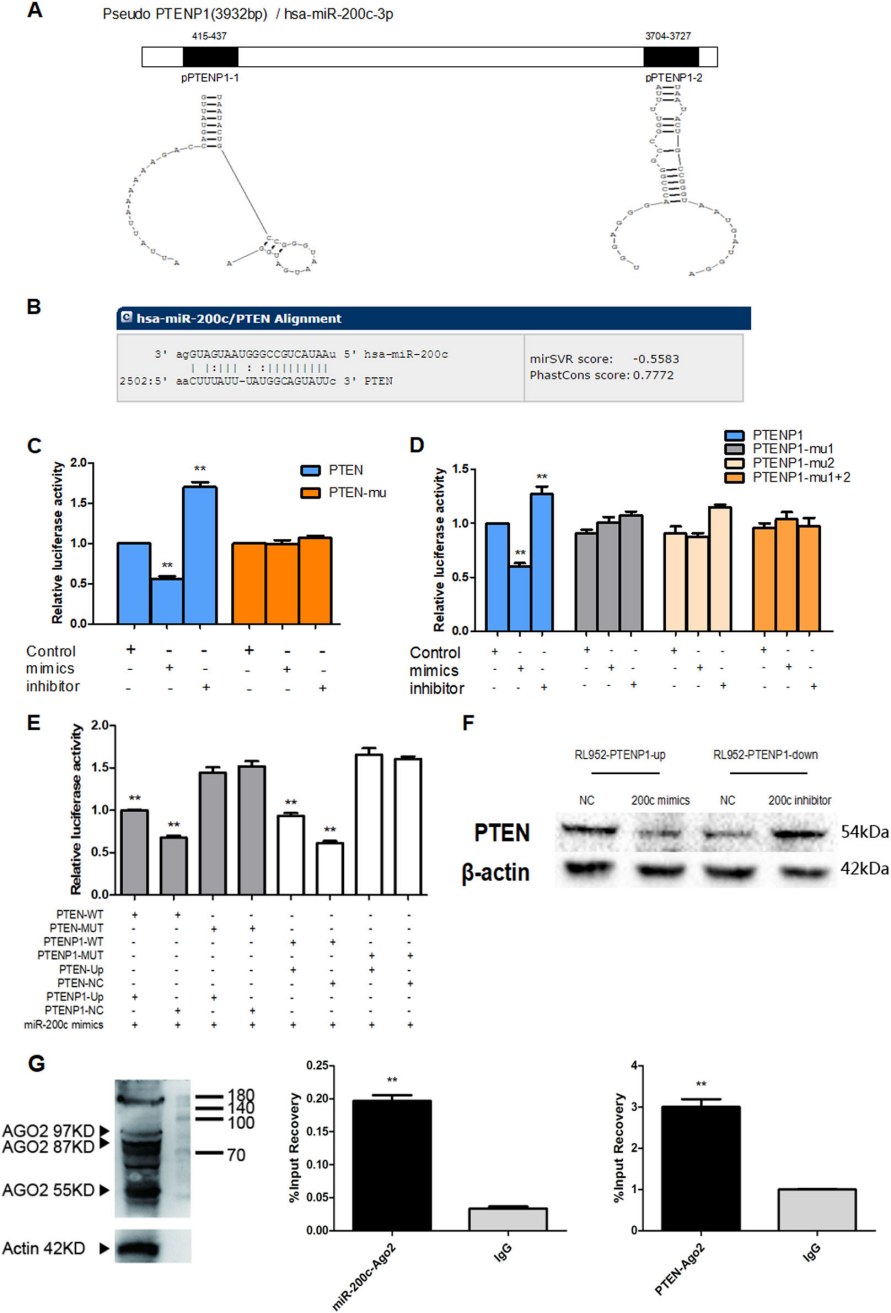
PTENP1 and PTEN are target genes of miR-200c. **a**, **b** http://www.microrna.org and ClustalW2/RNA structure was predicted that PTEN or PTENP1 have bound to miR-200c directly. **c** miR-200c mimics were co-transfected with the PTEN wt 3′UTR or mt 3′UTR. Luciferase activities were used to detect the effect on PTEN expression at 48 h after transfection. **d** miR-200c mimics were co-transfected with the PTENP1 wt 3′UTR, mt 3′UTR-1, mt 3′UTR-2, or mt 3′UTR-1+2 as indicated. Luciferase activities were used to detect the effect on PTENP1 expression at 48 h after transfection. **e** Co-transfection dual-luciferase reporter by transfecting RL-952 cells with miR-200c mimics to verify the interaction between the PTENP1, PTEN, and miR-200c. **f** MiR-200c and PTENP1 palsmid co-transfection with RL-952 cells to detected the change of PTEN protein in western blot. **g** The interaction with microRNA-200c and PTEN was assayed using the method of AGO2-RIP. PTEN can combined with Ago2 protein as well as miR-200c to form an RNA-induced silencing complex. PTEN-Ago2 were significantly higher than negative control group (IgG). All results are presented as the mean of triplicate assays. **P < 0.01

The binding sites of PTENP1, PTEN, and miR-200c were found by bioinformatics prediction on the website (http://www.microrna.org) or ClustalW2 and RNA structure. The details of which are shown in Fig. 3a, b. To further study the binding mechanism between miR-200c and PTEN or PTENP1, we constructed wild-type (WT) and mutant (Mut) luciferase reporter vectors according to the binding sites. The dual-luciferase reporter results are as follow, the results of the PTEN binding-site test showed that upregulation of miR-200c could cause a decrease in the relative luciferase activity of PTEN. In contrast, downregulation of miR-200c could increase the PTEN activity. It verified that PTEN was the target gene of miR-200c (Fig. 3c). Similarly to PTEN, miR-200c mimics could cause a decrease of PTENP1 activity, and the miR-200c inhibitor could increase the activity of PTENP1, therefore, PTENP1 was also the target gene of miR-200c (Fig. 3d).

Subsequently, we used a co-transfection dual-luciferase reporter assay to verify the interaction between the PTENP1, PTEN, and miR-200c. The results showed that when the expression of PTENP1 was upregulated, the decrease in the luciferase activity of the PTEN reporter plasmid caused by the miR-200c mimic was reversed. Similarly, when the expression of PTEN was upregulated, the decrease in the luciferase activity of the PTENP1 reporter plasmid caused by miR-200c mimics was also reversed (Fig. 3e). Next, we used RL-952 cells to co-transfect miR-200c and PTENP1 plasmid. The results showed that when the expression of miR-200c and PTENP1 was upregulated, the positive regulation of PTENP1 on PTEN was reversed. Similarly, when the expression of miR-200c and PTENP1 was downregulated, the positive regulation of PTENP1 on PTEN was also reversed (Fig. 3f). All these findings suggested that both PTEN and the PTENP1 pseudogene have a common miR-200c binding site, and the expression of PTENP1 and PTEN is dependent on miR-200c. The results indicated that PTEN could be competitively regulated by PTENP1 and miR-200c.

Moreover, we further verified their relationship using the RIP assay. The results showed that the expression level of miR-200c-Ago2 and PTEN-Ago2 were significantly higher than that of the negative control (IgG). RNA immunoprecipitation suggests that Ago2 can be combined with PTEN as well as miR-200c to form an RNA-induced silencing complex (Fig. 3g).

### E2 adjust competing regulation of PTENP1-miR-200c to PTEN through estrogen receptor alpha

**Fig. 4:**
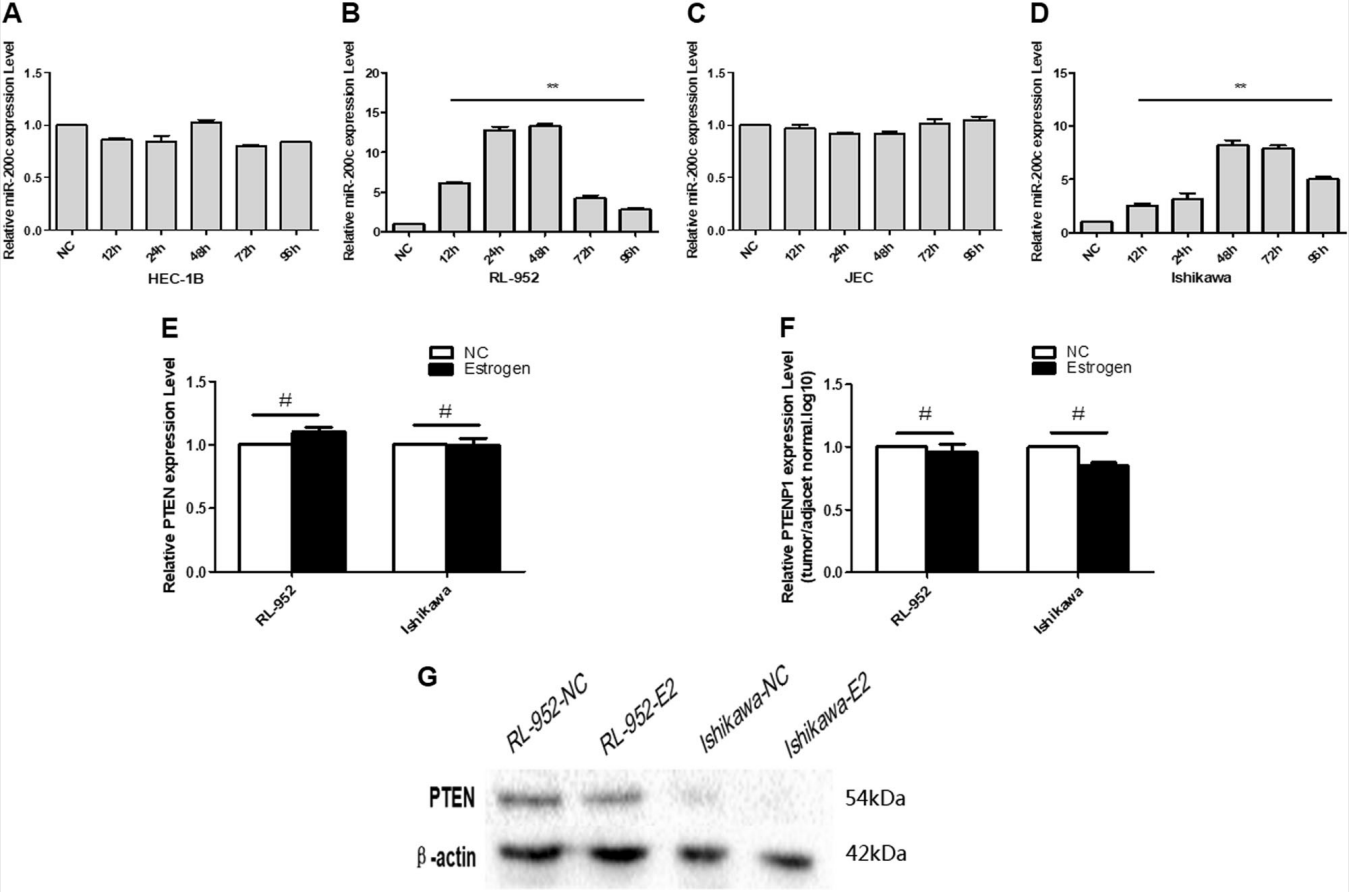
E2 could adjust the expression of miR-200c and PTEN in EEC cell lines. **a**, **b** After induced by estrogen for 12, 24, 48, 72, and 96 h in RL-952 cell lines, the expression of miR-200c in RL-952 and Ishikawa cell lines increased but HEC-1B and JEC cell lines was no significant change. **c**, **d** The RNA level of PTEN and PTENP1 was no significant change with RL-952 and Ishikawa cell lines after E2-induced for 48 h. **g** Western blot showed that the expression of PTEN protein in RL-952 cells was downregulated after E2-induced for 48 h. The results are presented as the mean of triplicate assays, with S.D. **P < 0.01

To research estrogen’s role in the regulation of PTEN, E2 was induced in EEC cell line culture. First, four EEC cell lines (HEC-1B, RL-952, JEC and Ishikawa) were induced by stimulation with 0.1 µm of E2. The expression of miR-200c in the RL-952 and Ishikawa cell lines was increased after E2 induction, and 48 h was the best induction time, but HEC-1B and JEC did not change significantly (Fig. 4a, d). On the other hand, the RNA expression of PTENP1 and PTEN was no change (Fig. 4e, f), but the PTEN protein was significantly decreased when RL-952 was induced by E2 for 48 h, indicating that E2 may downregulate the expression of the PTEN protein probably by induction or upregulation of miR-200c, and miR-200c dropping PTEN protein through posttranscriptional control (Fig. 4g).

**Fig. 5:**
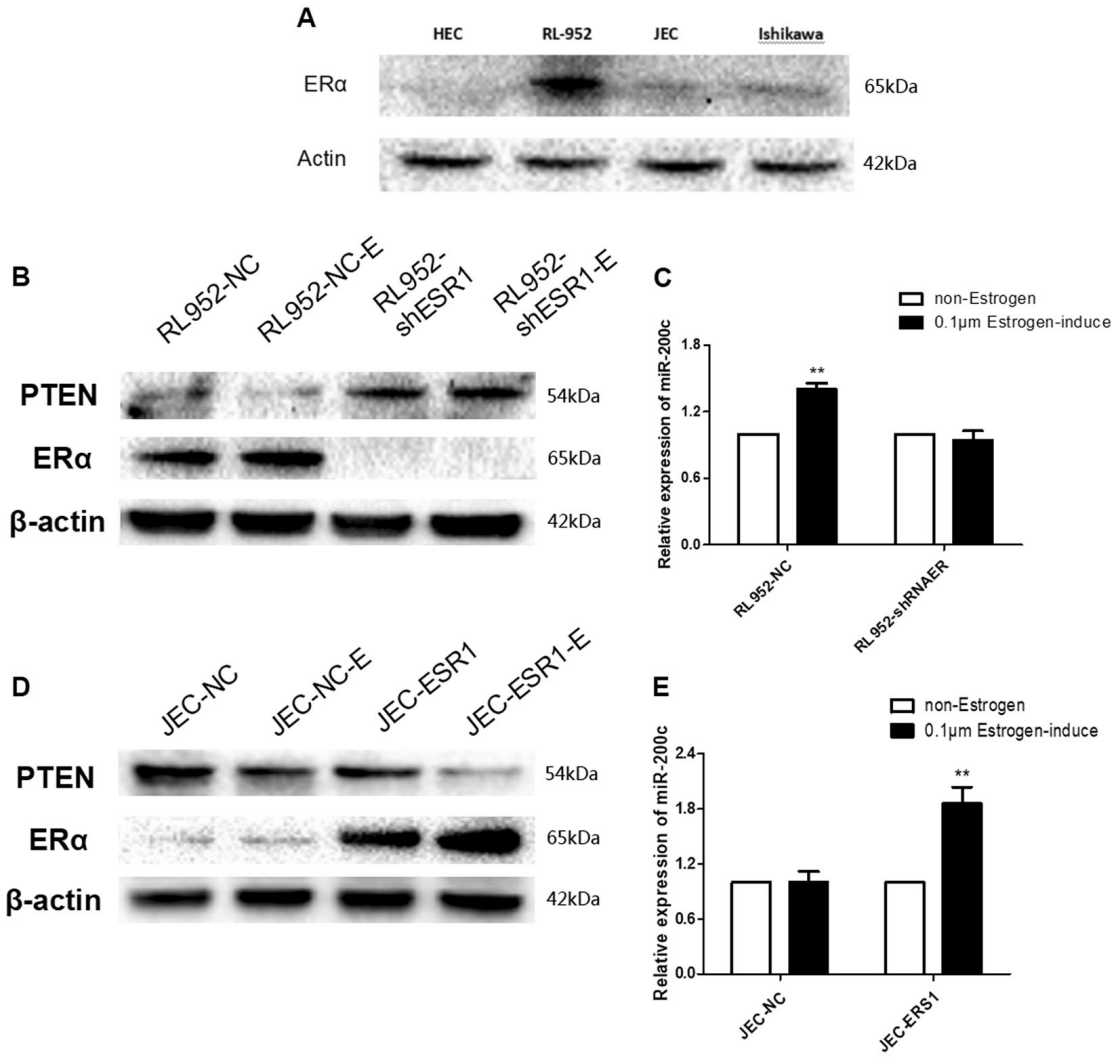
E2 affected the expression of miR-200c and PTEN proteins through estrogen receptor Alpha. **a** The expression of estrogen receptor Alpha in four EEC cell lines. **b**, **c** The upregulation of miR-200c and downregulation of PTEN protein were disappeared after E2-induced for 48 h when stable knockdown the ESR1 in RL-952 cell lines. **d**, **e** The expression of miR-200c was increased and PTEN protein was downregulated after E2-induced for 48 h when stable overexpression the ESR1 in JEC cell lines. All results are presented as the mean of triplicate assays, with S.D. **P < 0.01

To understand how E2 adjusted miR-200c and PTEN, We first observed two EEC cell lines HEC-1B and JEC which did not respond to E2 induction, the estrogen receptor 1 (ESR1) levels were negative or weakly expressed (Fig. 5a). Then we constructed ESR1 overexpression stable EEC cell lines pER-JEC and knockdown one shER-RL-952. It was found that when E2 induced in shER-RL-952, the primarily upregulated miR-200c and downregulated PTEN were reversed in shER-RL-952 cells (Fig. 5b–c). In contrast, when E2 induced in pER-JEC, miR-200c level was found increased and the PTEN protein decreased (Fig. 5d–e). These results indicated that E2 may directly adjust the regulation of PTENP1, miR-200c, and PTEN by ERα.

### E2 promotes cell proliferation, migration, invasion, and anti-apoptosis by upregulating miR-200c in EEC cells

To determine whether the upregulation of miR-200c through E2-induction influences EEC proliferation, migration and invasion, E2-induced RL-952 cells were simultaneously transfected with miR-200c mimic or inhibitor.

**Fig. 6:**
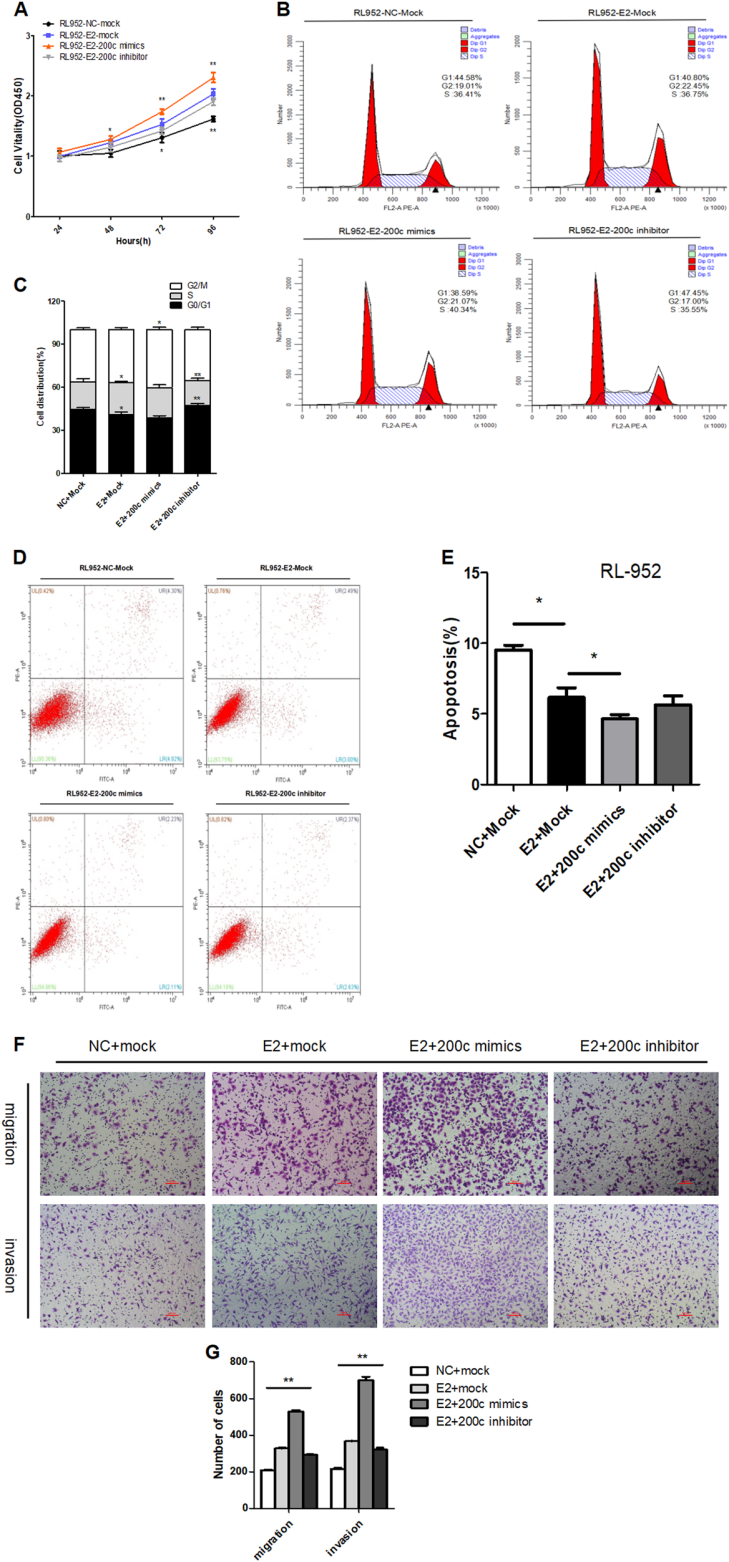
E2 promoted cell proliferation, migration, invasion, and anti-apoptosis by upregulating miR-200c. **a** CCK-8 assay showing cell proliferation in RL-952 cells that were transfected with miR-200c mimics or inhibitor combine with E2-induced for 48 h. **b**, **c** Flow cytometry analysis showed cell cycle in RL-952 cells that were transfected with miR-200c mimics or inhibitor combine with E2-induced for 48 h. **d**, **e** FACS analysis showing apoptosis in RL-952 cells that were transfected with miR-200c mimics or inhibitor combine with E2-induced for 48 h. **f**, **g** Migration and invasion assay in RL-952 EEC cells that were transfected with the miR-200c mimics or inhibitor combine with E2-induced for 24 h. Cells were evaluated at 16 h after transfection (×200 magnification). The results are shown as the mean ± SEM from three independent experiments (*P < 0.05, **P < 0.01)

The results of CCK-8 assay indicated that E2 could increase cell proliferation when E2 and miR-200c mimics were concurrently added, causing a much higher proliferative rate. Accordingly, when the expression of miR-200c is inhibited concurrently, the cell activity was lower (Fig. 6a). The results suggested that E2 could promote proliferation relying on miR-200c level in EEC cells. Similarly to the CCK-8 assay, flow cytometry analysis showed that E2 could promote cell growth and increase the proportion of cells in the S phase, while cell growth was faster after transfection of miR-200c mimic. However, after transfection of the miR-200c inhibitor, cell livability and the proportion of cells in S phase dropped, while the proportion in the G1 phase increased (Fig. 6b, c). Additionally, FACS analysis showed that E2 could retard apoptosis, while miR-200c mimics transfected but did not produce a significant difference in the apoptosis rate compared to the transfected miR-200c inhibitor (Fig. 6d, e).

The results of the cell migration and invasion showed that E2 induction could promote cell migration and invasion, which were normally motivated by miR-200c. We also transfected miR-200c inhibitor with induced E2 and observed that the downregulation of miR-200c could reverse the migration and invasion activity (Fig. 6f, g).

These results indicate that E2 may modulate cell proliferation, migration and invasion relying on the expression of miR-200c.

### E2 activates the PI3K-AKT pathway by upregulating miR-200c expression in EEC cells

**Fig. 7:**
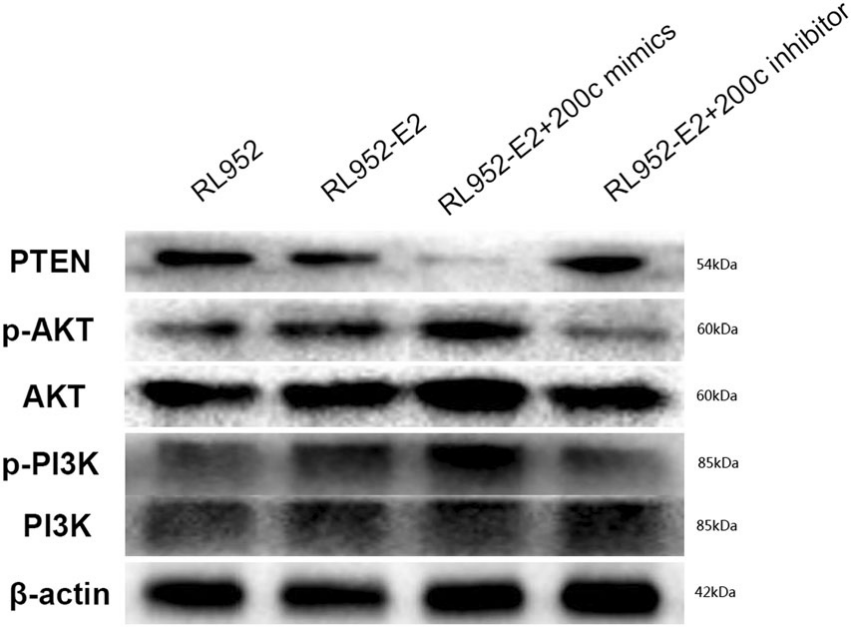
E2 activated the PI3K-AKT-mTOR pathway by upregulating miR-200c expression in EEC cells. Western blot showed that phosphorylated AKT and PI3K protein could be upregulated or downregulated in RL-952 cells by tranfecting miR-200c mimic or inhibitor combine with E2-induced 48 h

The above experiment demonstrated that estrogen could downregulate the expression of PTEN by upregulating miR-200c. At the same time, we found that phosphorylated AKT and PI3K protein could be upregulated by E2-induction in RL-952 cells. Moreover, the effect of E2 was inhibited after transfection with miR-200c inhibitors (Fig. 7). This experiment further demonstrates that estrogen could play a role through miR-200c to downregulate PTEN and then indirectly activate the PI3K-AKT pathway.

## Discussion

The development of EEC is the result of multiple factors and the participation of different genes. A major risk factor that is widely accepted is the lack of progesterone antagonism, leading to a relative excess of estrogen^31,32^. Estrogen can affect biological behavior by genetical or non-genetical combing with estrogen receptor (ER) alpha or beta in vivo^33,34^. In addition, another feature of EEC is the mutation or loss of PTEN, which is closely related to tumor development in terms of cell growth, proliferation, migration, and differentiation^35^. An accumulating amount of evidence indicated that increased estrogen and decreased PTEN can participate and play opposite roles in the PI3K-AKT-mTOR pathway, and estrogen can inhibit PTEN. Through sequence analysis, the mutation of PTEN was found in a variety of cancers^36^, but the mechanism is not understood.

In recent years, a large number of studies on miRNAs have shown that they play an important role in biological activities. MiRNAs can promote or inhibit the expression of their proteins by binding to the target coding genes to play a biological function^37^, including the pathogenesis of cancer^38^. However, this binding is not limited to encoding RNA, they can also bind non-coding RNAs^39–41^. In our previous studies, miR-200c could target and bind lncRNA MALAT1 to downregulate the MALAT1 expression and inhibit the epithelial-mesenchymal transition (EMT) of EEC^24^. However, the upregulated miR-200c in EEC make us wonder if miR-200c has another role that has yet to be explored. Additionally, the relationship between miR-200c and PTEN has rarely been reported.

On the other hand, increasing amounts of pseudogenes have been shown to be involved in the transcription process^42,43^, or regulatory function^44^, and even participated in cancer pathogenesis^45,46^. As the only PTEN pseudogene, PTENP1 is highly homologous to PTEN and has an almost 95% sequence similarity with PTEN, thus having many shared miRNA binding sites. Poliseno found that PTENP1 could compete for miRNA affect the expression of PTEN^17^. Decreasing the expression of PTENP1 could lead to the release of miRNAs, which in turn binds to PTEN, leading to a drop in PTEN protein expression. So far, miR-19b, miR-20a, miR-21, miR-26a, and miR-214 are involved in PTENP1-miRNA-PTEN regulation and have been reported in some carcinomas, such as prostate carcinoma^17,18^, renal cell carcinoma^19^, and so on^46^. However, there are no related reports found in EEC.

Based on the above research, we collected 40 cases of EEC samples and used qRT-PCR to detect the expression of PTENP1, miR-200c, and PTEN. The results showed that miR-200c was highly expressed, while PTENP1 and PTEN declined compared with the adjacent normal endometrium, there was an opposite relationship between miR-200c compared with PTENP1 and PTEN. Additionally, the lost level of PTENP1 was more severe than PTEN. At the same time, according to bioinformatics analysis, we found that miR-200c binds to PTEN and PTENP1, respectively. Therefore, we began to study the relationship between PTENP1, PTEN, and miR-200c and their relationship with estrogen.

The study demonstrated that miR-200c negatively regulates the expression of PTENP1 and PTEN, whereas PTENP1 positively regulates PTEN and negatively regulates miR-200c using qRT-PCR, western blots and a series of transfection experiments. Then, using dual-luciferase reporters and RIP assays, it was found that miR-200c could bind to PTEN and PTENP1, respectively, and the regulation of PTENP1 on PTEN relies on the expression of miR-200c. PTENP1-miR-200c-PTEN forms a network of negative feedback loops. We hypothesized that the overexpression of miR-200c is due to the mutation of PTENP1, whereas excess miR-200c binds to the tumor suppressor gene PTEN, resulting in the loss or mutation of the latter in EECs.

As we know, estrogen plays an important role in the occurrence of EEC. The expression of miR-200c in EEC cells was increased, and the expression of PTEN protein decreased when inducting E2. However, this phenomenon declined when we stably knocked down ESR1. CCK-8, flow cytometry analysis and invasion assays found that estrogen can promote the development of EEC cells relies on miR-200c. The high expression of miR-200c can accelerate the induction of estrogen on EEC cell lines, stimulate proliferation and inhibit the apoptosis activity. These results suggest that estrogen can upregulate the expression of miR-200c and further affect feedback of PTENP1-miR-200c-PTEN. This results in the downregulation of PTEN protein expression, which leads to the phosphorylation of the AKT protein and activation of the PI3K/AKT pathway, inhibiting EEC cell apoptosis and promoting proliferation in EEC.

Briefly, in this study, we demonstrated that the feedback loop of PTENP1-miR-200c-PTEN in EEC can be regulated by estrogen, and this regulation is relied on combination of estrogen and ER. However, further study is still necessary on how estrogen leads to miR-200c expression and research into downstream pathways. Additionally, it will be the focus of this research in the future.

## Electronic supplementary material

Supplementary figure1

Supplementary figure2

Supplementary figure3

Supplementary table1

Supplementary table2

Supplementary figure legends

## References

[1] Siegel RL, Miller KD, Jemal A (2017). Cancer statistics, 2017. CA Cancer J. Clin..

[2] Chen W (2016). Cancer statistics in China, 2015. CA Cancer J. Clin..

[3] Yeramian A (2013). Endometrial carcinoma: molecular alterations involved in tumor development and progression. Oncogene.

[4] McConechy MK (2012). Use of mutation profiles to refine the classification of endometrial carcinomas. J. Pathol..

[5] Bozdog Ouml, Atasoy P, Erekul S, Bozdoğan N, Bayram M (2002). Apoptosis-related proteins and steroid hormone receptors in normal, hyperplastic, and neoplastic endometrium. Int. J. Gynecol. Pathol..

[6] Wik E (2013). Lack of estrogen receptor-α associated with epithelial–mesenchymal transition and PI3K alterations in endometrial carcinoma. Clin. Cancer Res..

[7] Guzeloglu-Kayisli O (2003). Regulation of PTEN (phosphatase and tensin homolog deleted on chromosome 10) expression by estradiol and progesterone in human endometrium. J. Clin. Endocrinol. Metab..

[8] Lagarda H, Catasus L, Arguelles R, Matias-Guiu X, Prat J (2001). K-ras mutations in endometrial carcinomas with microsatellite instability. J. Pathol..

[9] Mutter GL, Lin M, Fitzgerald JT, Kum JB, Eng C (2000). Changes in endometrial PTEN expression throughout the human menstrual cycle. J. Clin. Endocrinol. Metab..

[10] Waite KA, Eng C (2002). Protean PTEN: form and function. Am. J. Hum. Genet..

[11] Eng C (2003). PTEN: one gene, many syndromes. Hum. Mutat..

[12] Bussaglia E, Del Rio E, Matias-Guiu X, Prat J (2000). PTEN mutations in endometrial carcinomas: a molecular and clinicopathologic analysis of 38 cases. Hum. Pathol..

[13] Ali IU (2000). Gatekeeper for endometrium: the PTEN tumor suppressor gene. J. Natl. Cancer Inst..

[14] Lian Z, De Luca P, Di Cristofano A (2006). Gene expression analysis reveals a signature of estrogen receptor activation upon loss ofPten in a mouse model of endometrial cancer. J. Cell. Physiol..

[15] Vilgelm A (2006). Akt-mediated phosphorylation and activation of estrogen Receptor A is required for endometrial neoplastic transformation in Pten+/- mice. Cancer Res..

[16] Joshi A (2012). Endometrial tumorigenesis in Pten+/− mice is independent of coexistence of estrogen and estrogen receptor α. Am. J. Pathol..

[17] Poliseno L (2010). A coding-independent function of gene and pseudogene mRNAs regulates tumour biology. Nature.

[18] Dahia PL (1998). A highly conserved processed PTEN pseudogene is located on chromosome band 9p21. Oncogene.

[19] Yu G (2014). Pseudogene PTENP1 functions as a competing endogenous RNA to suppress clear-cell renal cell carcinoma progression. Mol. Cancer Ther..

[20] Ioffe YJ, Chiappinelli KB, Mutch DG, Zighelboim I, Goodfellow PJ (2012). Phosphatase and tensin homolog (PTEN) pseudogene expression in endometrial cancer: a conserved regulatory mechanism important in tumorigenesis?. Gynecol. Oncol..

[21] Tang H (2013). miR-200b and miR-200c as prognostic factors and mediators of gastric cancer cell progression. Clin. Cancer Res..

[22] Yuan D (2017). MiR-200c inhibits bladder cancer progression by targeting lactate dehydrogenase A. Oncotarget.

[23] Teng Y (2016). miRNA-200a/c as potential biomarker in epithelial ovarian cancer (EOC): evidence based on miRNA meta-signature and clinical investigations. Oncotarget.

[24] Li Q (2016). Disrupting MALAT1/miR-200c sponge decreases invasion and migration in endometrioid endometrial carcinoma. Cancer Lett..

[25] Xiong H (2016). A multi-step miRNA-mRNA regulatory network construction approach identifies gene signatures associated with endometrioid endometrial carcinoma. Genes-Basel.

[26] Wu W, Lin Z, Zhuang Z, Liang X (2009). Expression profile of mammalian microRNAs in endometrioid adenocarcinoma. Eur. J. Cancer Prev..

[27] Lee J (2011). The expression of the miRNA-200 family in endometrial endometrioid carcinoma. Gynecol. Oncol..

[28] Brabletz S, Brabletz T (2010). The ZEB/miR-200 feedback loop--a motor of cellular plasticity in development and cancer?. EMBO Rep..

[29] Hill L, Browne G, Tulchinsky E (2013). ZEB/miR-200 feedback loop: at the crossroads of signal transduction in cancer. Int. J. Cancer.

[30] Gregory PA (2008). The miR-200 family and miR-205 regulate epithelial to mesenchymal transition by targeting ZEB1 and SIP1. Nat. Cell Biol..

[31] Hecht JL, Mutter GL (2006). Molecular and pathologic aspects of endometrial carcinogenesis. J. Clin. Oncol..

[32] Potischman N (1996). Case-control study of endogenous steroid hormones and endometrial cancer. J. Natl. Cancer Inst..

[33] Sun Y (2014). Estradiol promotes pentose phosphate pathway addiction and cell survival via reactivation of Akt in mTORC1 hyperactive cells. Cell Death Dis..

[34] Liang J, Shang Y (2013). Estrogen and cancer. Annu. Rev. Physiol..

[35] Lu KH, Daniels M, Broaddus RR (2008). Germline PTEN mutations as a cause of early-onset endometrial cancer. J. Clin. Oncol..

[36] Salmena L, Carracedo A, Pandolfi PP (2008). Tenets of PTEN tumor suppression. Cell.

[37] Lai EC (2005). miRNAs: whys and wherefores of miRNA-mediated regulation. Curr. Biol..

[38] Rupaimoole R, Calin GA, Lopez-Berestein G, Sood AK (2016). miRNA deregulation in cancer cells and the tumor microenvironment. Cancer Discov..

[39] Braconi C (2011). microRNA-29 can regulate expression of the long non-coding RNA gene MEG3 in hepatocellular cancer. Oncogene.

[40] Wang P (2015). Long non-coding RNA CASC2 suppresses malignancy in human gliomas by miR-21. Cell. Signal..

[41] Zhang Z (2013). Negative regulation of lncRNA GAS5 by miR-21. Cell Death Differ..

[42] Pei B (2012). The GENCODE pseudogene resource. Genome Biol..

[43] Kalyana-Sundaram S (2012). Expressed pseudogenes in the transcriptional landscape of human cancers. Cell.

[44] Hawkins PG, Morris KV (2014). Transcriptional regulation of Oct4 by a long non-coding RNA antisense to Oct4-pseudogene 5. Transcription.

[45] Johnsson P, Morris KV, Grandér D (2014). Pseudogenes: a novel source of trans-acting antisense RNAs. Methods Mol. Biol..

[46] Poliseno L (2011). Deletion of PTENP1 pseudogene in human melanoma. J. Invest. Dermatol..

