# Supplementary figures and images for: Estrogen affects the negative feedback loop of PTENP1-miR200c to inhibit PTEN expression in the development of endometrioid endometrial carcinoma
Source: Cell Death Dis. 2018 Dec 18;10(1):4. doi: 10.1038/s41419-018-1207-4 (PMC6315040; doi:10.1038/s41419-018-1207-4)

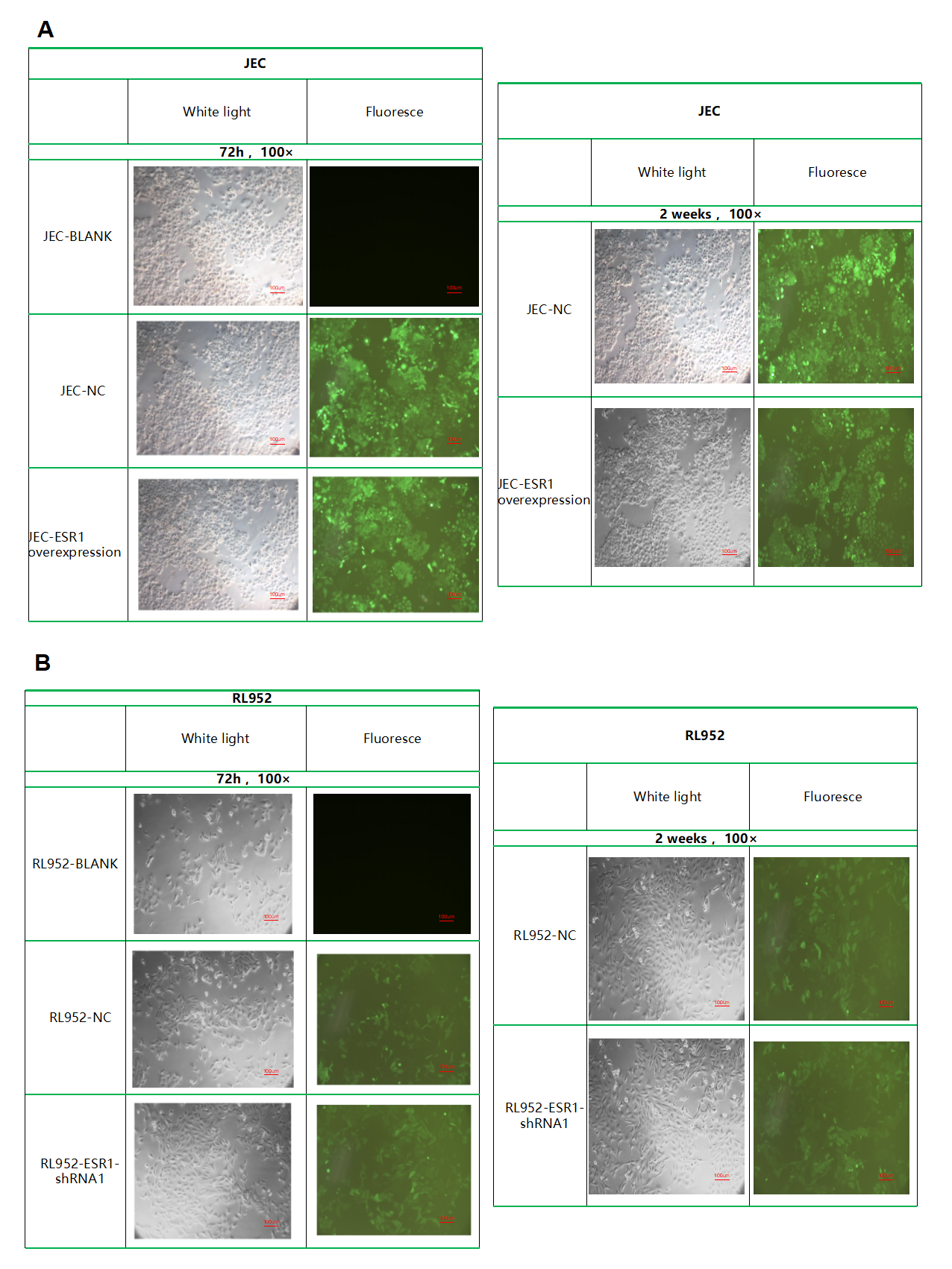

Supplement: Supplementary file 1 — Supplementary figure1 [file 41419_2018_1207_MOESM1_ESM.tif]

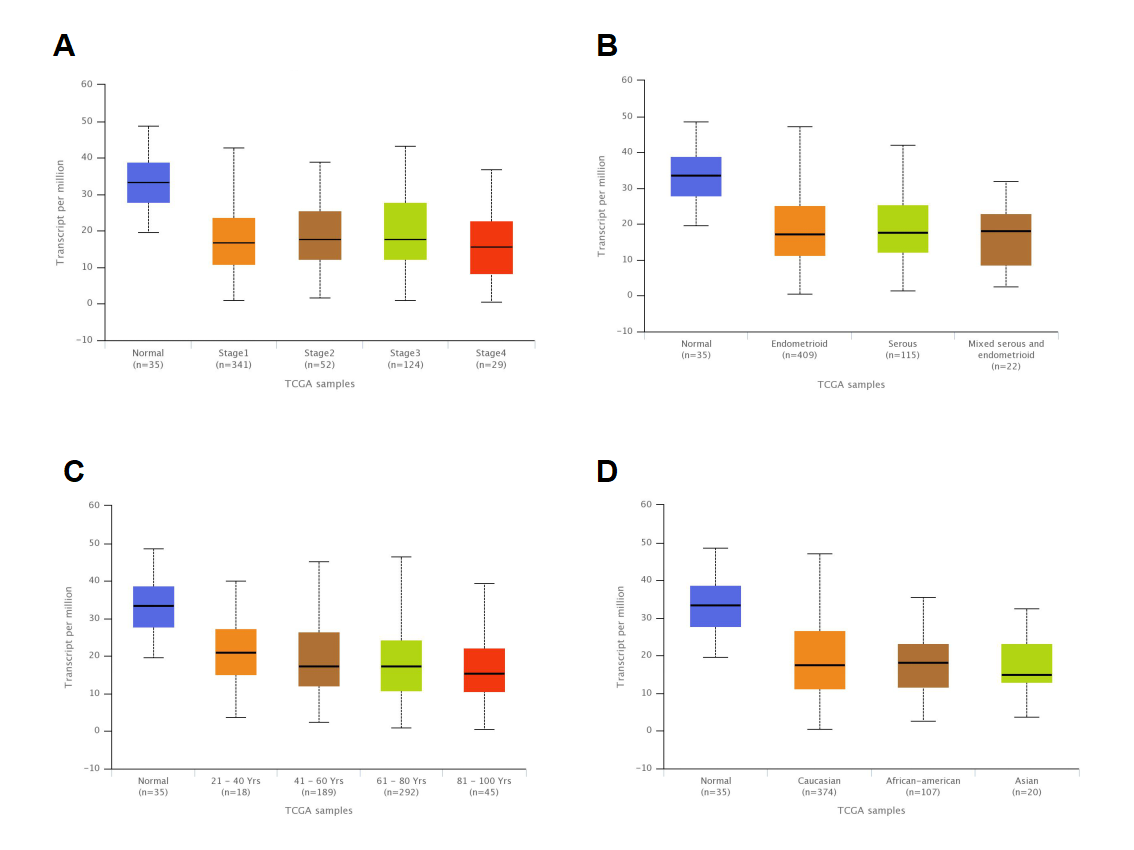

Supplement: Supplementary file 2 — Supplementary figure2 [file 41419_2018_1207_MOESM2_ESM.tif]

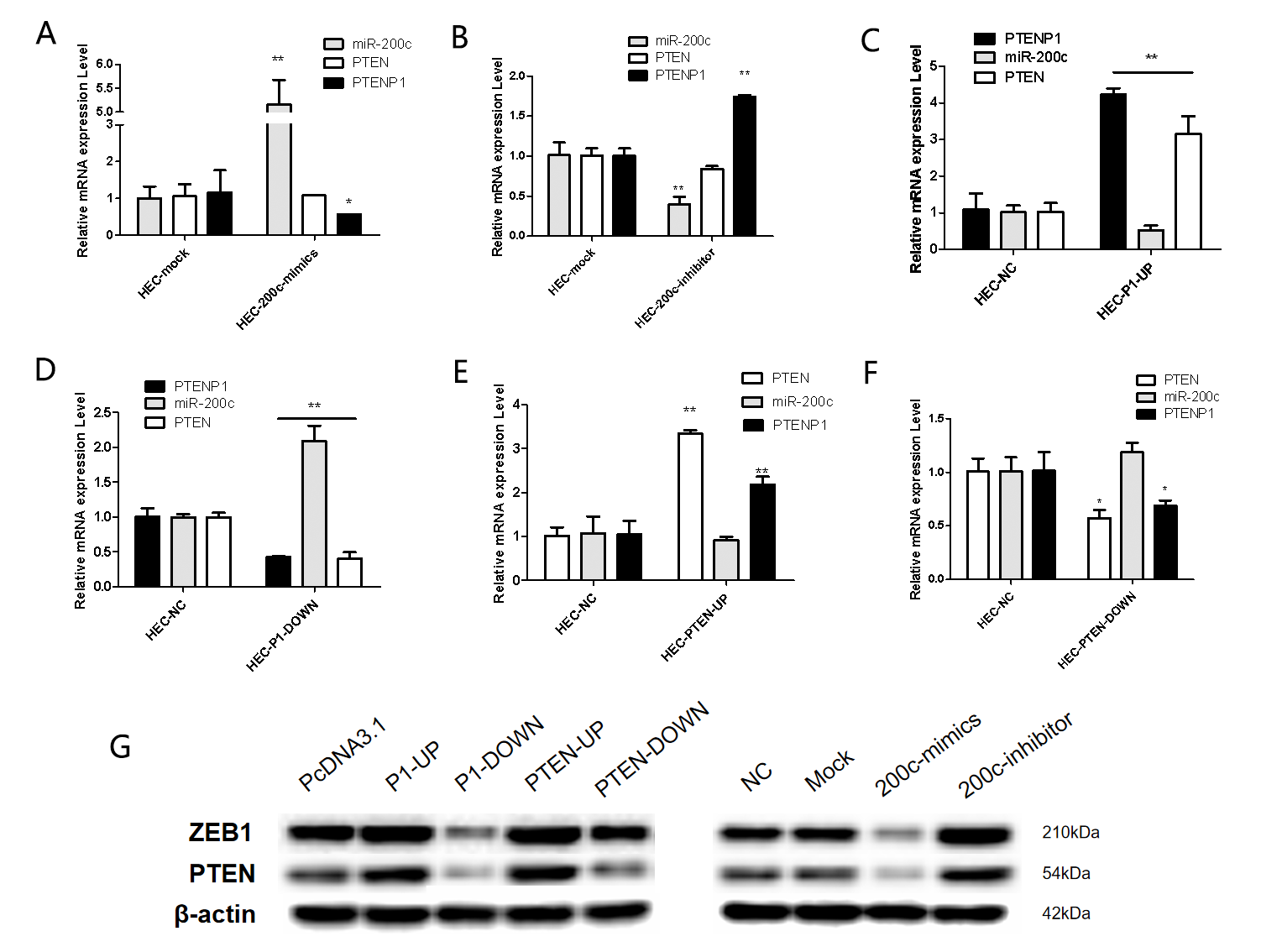

Supplement: Supplementary file 3 — Supplementary figure3 [file 41419_2018_1207_MOESM3_ESM.tif]
