# Supplementary material for: Estrogen affects the negative feedback loop of PTENP1-miR200c to inhibit PTEN expression in the development of endometrioid endometrial carcinoma
Source: Cell Death Dis. 2018 Dec 18;10(1):4. doi: 10.1038/s41419-018-1207-4 (PMC6315040; doi:10.1038/s41419-018-1207-4)
Supplement: Supplementary file 5 — Supplementary table2 [file 41419_2018_1207_MOESM5_ESM.pptx]

## Slide 1
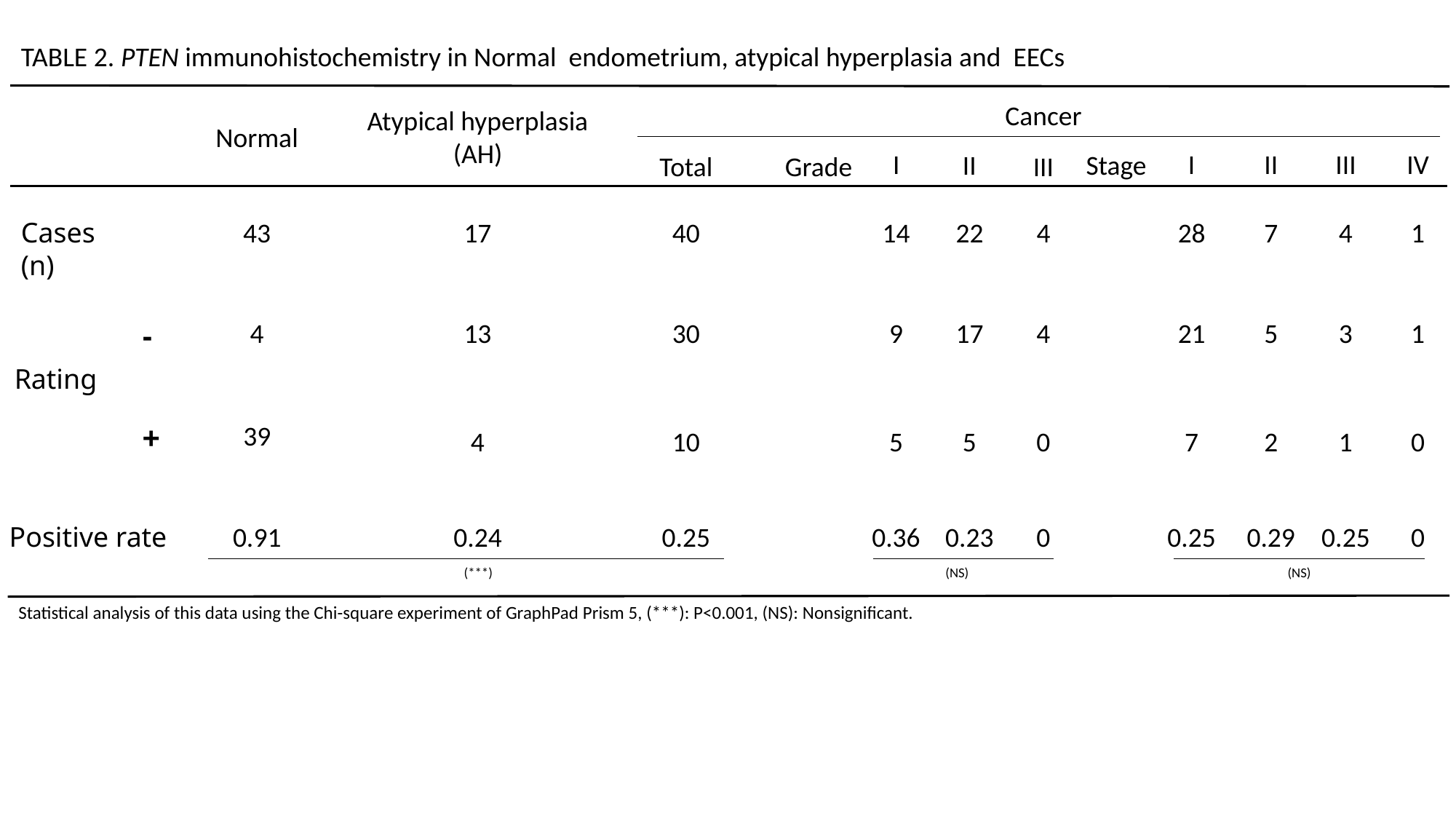

TABLE 2. PTEN immunohistochemistry in Normal endometrium, atypical hyperplasia and EECs
Cancer
Atypical hyperplasia
(AH)
Normal
I
I
II
III
IV
II
Stage
Total
Grade
III
Cases (n)
43
17
40
14
22
4
28
7
4
1
-
4
13
30
9
17
4
21
5
3
1
Rating
+
39
4
10
5
5
0
7
2
1
0
Positive rate
0.91
0.24
0.25
0.36
0.23
0
0.25
0.29
0.25
0
(***)
(NS)
(NS)
Statistical analysis of this data using the Chi-square experiment of GraphPad Prism 5, (***): P<0.001, (NS): Nonsignificant.
