# Supplementary material for: Estrogen affects the negative feedback loop of PTENP1-miR200c to inhibit PTEN expression in the development of endometrioid endometrial carcinoma
Source: Cell Death Dis. 2018 Dec 18;10(1):4. doi: 10.1038/s41419-018-1207-4 (PMC6315040; doi:10.1038/s41419-018-1207-4)
Supplement: Supplementary file 6 — Supplementary figure legends [file 41419_2018_1207_MOESM6_ESM.doc]

**Supplementary Figure 1**

JEC(A) and RL952(B) cells were infected by lentivirus respectively and screened two days and two weeks after the stable fluorescence photographs. Killing of cells that were not effectively infected by the addition and maintenance of 10 ug/ml puromycin, indicated that the stable cells were screened for the expected results.

**Supplementary Figure 2**

The expression of PTEN in 546 cases of endometrial carcinoma were analyzed by Ualcan database (http://ualcan.path.uab.edu/analysis.html). The original numbers were derived from ATCC database.

In endometrial cancer, PTEN mRNA was low expressed and with no significant difference in different clinical stages(A), histological type(B), ages(C) and race(D),P>0.05.

**Supplementary Figure 3**

The relationship between miR-200c, PTENP1 and PTEN in HEC-1B cell line.

A. PTENP1 was down-regulated by transfecting HEC1-B cells with miR-200c mimics, when PTEN RNA was not significant changed. B. PTENP1 was up-regulated in HEC1-B cells when miR-200c expression was inhibited, when PTEN RNA was not significantly changed.C. PTEN was up-regulated and miR-200c was down-regulated when PTENP1 expression was increased in HEC-1B cells. D. PTEN was down-regulated and miR-200c was up-regulated when PTENP1 expression was inhibited in HEC-1B cells. E-F. The expression of PTENP1 increased or decreased after PTEN over-expression or interference in HEC-1B cells, but there was no significant change in miR-200c expression. G. the expression of PTEN protein increased or decreased by transfecting HEC-1B cells with miR-200c-inhibitor of miR-200c-mimics.

**Supplementary Table1**

Detailed primer sequences used in the article.

**Supplementary Table2**

PTEN immunohistochemistry in Normal endometrium, atypical hyperplasia and EECs. The results are using chi-square experiment of GraphPad Prism 5 to analyzed and statistics, (***): P<0.001, (NS): Nonsignificant.
